# Mechanisms of cooperative cell-cell interactions in skeletal muscle regeneration

**DOI:** 10.1186/s41232-022-00234-6

**Published:** 2022-11-16

**Authors:** Hiroyuki Koike, Ichiro Manabe, Yumiko Oishi

**Affiliations:** 1 Department of Biochemistry & Molecular Biology, Nippon Medical School, 1-1-5 Sendagi, Bunkyo-ku, Tokyo 113-8602 Japan; 2 Department of Systems Medicine, Chiba University Graduate School of Medicine, 1-8-1 Inohana, Chuo-ku, Chiba 260-8670 Japan

**Keywords:** Skeletal muscle stem cells, Single-cell RNA sequencing, Macrophage, Tissue regeneration, Inflammation

## Abstract

Skeletal muscles have an extraordinary capacity to regenerate themselves when injured. Skeletal muscle stem cells, called satellite cells, play a central role in muscle regeneration via three major steps: activation, proliferation, and differentiation. These steps are affected by multiple types of cells, such as immune cells, fibro-adipogenic progenitor cells, and vascular endothelial cells. The widespread use of single-cell sequencing technologies has enabled the identification of novel cell subpopulations associated with muscle regeneration and their regulatory mechanisms. This review summarizes the dynamism of the cellular community that controls and promotes muscle regeneration, with a particular focus on skeletal muscle stem cells.

## Introduction

Skeletal muscle is an essential organ for metabolism and physical activity. It also has a high regenerative potential, with skeletal muscle stem cells (MuSCs), called satellite cells, playing a crucial role in its regeneration processes [1, 2]. Under normal conditions, MuSCs remain undifferentiated; however, when skeletal muscle is damaged, these cells become activated, start proliferating, and differentiate into myofibers, which are multinucleated fibrous myocytes [3]. Previous findings have shown that the sequence of activation, proliferation, and differentiation of MuSCs during muscle regeneration is highly affected by other cells, such as fibro-adipogenic progenitors (FAPs), vascular endothelial cells (ECs), and immune cells [4–7]. Furthermore, recent advances in single-cell sequencing technology have revealed the complex networks of cell populations that form the niche of muscle regeneration.

## MuSCs are activated in response to injury

MuSCs are essential for skeletal muscle homeostasis and regeneration. In uninjured muscle, MuSCs are mononuclear cells that reside between the plasma membrane and basement membrane of myofibers and are maintained in an undifferentiated state (G0, a reversible cell cycle arrest state) [8–10]. Undifferentiated MuSCs express the cell-type-specific transcription factor PAX7, whose expression is downregulated as MuSCs differentiate into myofibers during the course of muscle regeneration [11].

After being dissociated from the niche and cultured in vitro or following alterations in the niche environment in vivo, such as those induced by muscle injury, MuSCs deviate from their quiescent state and become activated [12], indicating the importance of the niche for maintaining the pool of MuSCs in a quiescent state. Indeed, it has been reported that surrounding cells, including vascular ECs and differentiated myofibers, provide signals that are involved in maintaining MuSCs in an undifferentiated state [13, 14]. In particular, signaling through the classical Notch pathway is crucial for maintaining quiescence [15–18]. ECs produce Notch ligand Dll4, which causes quiescence in MuSCs. Interestingly, MuSCs secrete VEGF-A, which promotes a close association of these cells with capillaries and suggests a reciprocal interaction between ECs and MuSCs [13]. Dll4 is also reported to be secreted by nascent muscle fibers, which triggers Notch 3 signaling and quiescence in associated MuSCs [14].

Muscle regeneration is achieved via the coordinated regulation of activation, proliferation, and differentiation of MuSCs [19, 20]. Upon injury, MuSCs are activated and migrate to the site of injury [21, 22]. Activated MuSCs are characterized by the expression of the transcription factors MYF5 and MYOD, as well as α7 integrin, and the myofilament protein, desmin. At the injury site, activated MuSCs proliferate and differentiate, which is characterized by the downregulation of *Pax7* and upregulation of myogenin and other muscle differentiation regulators [23–25]. Differentiated MuSC-derived myofibers regenerate skeletal muscle by further fusing with each other or with existing myofibers [26, 27]. Multiple cytokines such as IGF-1, FGF, HGF, PDGF, LIF, TNF-α, and IL-6 have been identified to promote the activation, proliferation, and differentiation processes of MuSCs, whereas TGF-β has been identified to inhibit these processes [9, 28]. In the following sections, we will focus on the intercellular interactions that regulate MuSC responses in muscle injury and regeneration.

The regenerative and developmental processes of muscle have some similarities and differences. MuSCs differentiate into muscle fibers during fetal or postnatal myogenesis as well as during regeneration after injury. The differentiation programs of MuSCs in both embryos and adults share some key transcriptional regulatory programs, such as regulation by muscle regulatory factors (MRFs), MYF5, MYOD, myogenin, and MRF4. However, the environmental cues that trigger MuSC activation and affect cell fates are different during embryonic muscle development and adult muscle regeneration; therefore, there are also some differences in the transcriptional mechanisms that regulate MuSCs. For instance, PAX3 plays a major role in early skeletal muscle formation in the embryo. In addition, while PAX7 is dispensable for embryonic muscle development in mice [29], it is essential for postnatal growth and muscle regeneration in the adult. Recent studies have also shown that there is diversity among MuSCs in adults. Whereas most adult MuSCs express only PAX7, expression of PAX3 in a subset of MuSCs confers higher resistance to environmental stress [30].

## Skeletal muscle injury models and muscle regeneration

Several experimental models for studying muscle regeneration and tissue repair in mice have been developed and involve the administration of myotoxic substances (cardiotoxin, notexin), chemicals (barium chloride), or physical damage (freeze injury) [31]. Although the spatiotemporal trajectory of the regenerative process in these muscle injury models may vary, presumably due to the magnitude of injury and differential effects on cells, such as immune cells, they all still generate initial myofiber damage and cell death, which elicits inflammation, followed by regeneration [31]. Much of the knowledge described in this review was obtained using these muscle injury models in mice and other animals.

Recently, in vitro models have been developed that reproduce components of the skeletal muscle tissue environment. In these models, artificial niches are created using ECMs, such as collagen and hydrogels [18], and by including internalized ECs and immune cells [32, 33] to successfully analyze the behaviors and functions of ECs and immune cells during regeneration. Indeed, these in vitro models have been useful for analyzing the changes in MuSCs as well as the interactions between specific cell types involved in skeletal muscle regeneration over time following damage by cardiotoxin or other agents.

## Neutrophils are involved in immune cell recruitment and MuSC activation

**Fig. 1:**
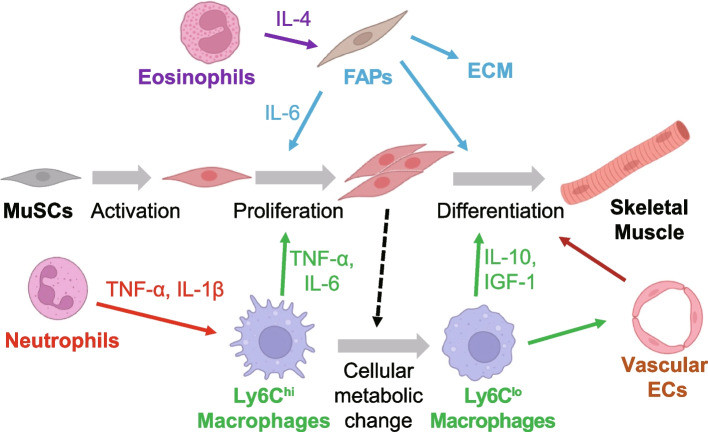
Cell-cell communication in skeletal muscle regeneration. MuSCs interact with immune cells, such as macrophages, as well as FAPs and vascular ECs to activate, proliferate, and differentiate into myofibers during skeletal muscle regeneration. These interactions are essential for proper skeletal muscle regeneration. Continuous changes in macrophage function are coordinated with the steps of myocyte regeneration. Soon after injury, Ly6C^hi^ cells predominate, which appear to include monocytes and macrophages. However, 2 to 3 days after injury, Ly6C^lo^ macrophages become predominant. MuSC: muscle stem cell; FAP: fibro-adipogenic progenitor; EC: endothelial cell; ECM: extracellular matrix. Created with BioRender.com

Upon injury, neutrophils are recruited by the neutrophil chemoattractants CXC-chemokine ligand 1 (CXCL1) and CC-chemokine ligand 2 (CCL2) produced by resident macrophages and by damage-associated molecular patterns (DAMPs), such as high-mobility group box 1 protein (HMGB1) [34]. Neutrophils are identified in skeletal muscle within 1–3 hours of muscle injury and induce the infiltration of many other types of immune cells [35] (Fig. 1). Since the depletion of neutrophils delays muscle regeneration after injury in mice [36], these cells are thought to play important roles in muscle regeneration. When the number of neutrophils reaches its peak (6–24 h after injury), MuSCs are observed to be activated and they express MYF5 and MYOD. This suggests the involvement of neutrophils in MuSC activation; however, direct interactions between neutrophils and MuSCs have not been fully validated.

## Macrophages promote proliferation and differentiation of MuSCs

Macrophages play vital roles not only in inflammation but also in tissue development, homeostasis, and regeneration [37–39]. Treatment with clodronate-containing liposomes, which induces apoptosis specifically in macrophages, was shown to cause impaired muscle regeneration in mice [40], indicating that macrophages are indispensable for muscle regeneration. After skeletal muscle injury, macrophages are recruited by cytokines secreted by neutrophils that have already infiltrated the injury site [5, 41] (Fig. 1). In the early phase of the injury response, Ly6C^hi^CCR2^+^ inflammatory monocytes are recruited and primarily differentiate into Ly6C^hi^ macrophages [42]. Systemic deletion of either *Ccr2* or *Cx3cr1* in mice was shown to result in impaired muscle regeneration and prolonged inflammation [43–45], suggesting that signaling through CCR2 and CX3CR1 are important for macrophage migration to the injury site. CCR2 ligand, CCL2/MCP-1, is expressed by damaged myocytes and resident macrophages and recruits monocytes/macrophages after injury [46].

According to recent single-cell RNA sequencing (scRNA-seq) time course results, Ly6C^hi^CCR2^+^ macrophages are the dominant cell population on the first day following cardiotoxin-induced skeletal muscle injury. On the third day, after the number of MuSCs in the skeletal muscle reaches its peak, the Ly6C^lo^ macrophage population takes over [47, 48]. Ly6C^hi^ and Ly6C^lo^ macrophages differentially contribute to skeletal muscle regeneration by supporting the proliferation and differentiation of MuSCs. Ly6C^hi^ macrophages secrete the inflammatory cytokines TNF-α and IL-6, which promote MuSC proliferation as well as the recruitment of additional macrophages [5, 49–51]. Indeed, systemic deletion of *Il-6* was shown to prevent macrophage recruitment to the site of injury, and muscle regeneration was impaired, with decreased MYOD and myogenin expression in myoblasts [52]. However, adoptive transfer of *Il-6*^+/+^ bone marrow cells into *Il-6*^−/−^ mice was able to rescue the impaired regeneration and improve MYOD and myogenin expression. Thus, IL-6 expression in monocytes/macrophages is critical for proper macrophage migration and myoblast proliferation during muscle regeneration.

Following injury, the phagocytosis of damaged tissue and cells promotes a shift in macrophages from their initial Ly6C^hi^CCR2^hi^ phenotype to a Ly6C^lo^CX3CR1^hi^ phenotype [3], which results in cells that produce high levels of insulin-like growth factor-1 (IGF-1) [5, 46]. IGF-1 has been shown to positively regulate muscle regeneration by promoting MuSC activation and differentiation [46, 53]. Aside from its effect on MuSCs, IGF-1 has also been shown to affect skeletal muscle fibers by activating the AKT pathway, which regulates both protein synthesis and degradation, resulting in skeletal muscle growth [54]. Moreover, in mice with conditional deletion of the *Igf-1* gene in myeloid cells, the rise of IGF-1 levels in injured muscle was suppressed, muscle inflammation was prolonged, and regeneration was hampered [55], suggesting that IGF-1 secreted by macrophages may also regulate macrophage activity. Indeed, transcriptomic analysis showed that *Igf-1* null macrophages exhibited inflammatory skewing and impaired activation of the reparative gene program. This dysregulation of macrophage function may contribute to the prolonged inflammation observed in response to injury in myeloid-specific *Igf-1*^−/−^ mice.

In addition to IGF-1, IL-10 and TGF-β are also released by Ly6C^lo^ macrophages in muscle, and their levels rise during the regeneration process, three days after damage occurs [35, 49, 56]. Expression of IL-10 increases during the transition from acute inflammation to the resolution phase [57, 58]. Systemic ablation of *Il-10* was shown to upregulate the early expression of inflammatory genes, such as *Il-6* and *Ccl2*, in skeletal muscle tissue on day 1 post-injury, while the upregulation of *Cd163* and *Arg1*, which mark macrophages in the inflammatory resolution and regeneration phase, was suppressed. In addition, in *Il-10*^−/−^ mice, the expression of muscle differentiation markers (e.g., myogenin) was temporally dysregulated, and muscle regeneration and tissue repair were greatly slowed after injury. These data show that IL-10 plays a role in regulating the switch of muscle macrophages from a pro-inflammatory to a pro-resolution phenotype in injured muscle, and that this transition is necessary for time-dependent myocyte differentiation and regeneration of muscle.

TGF-β1 is a multifunctional cytokine that is involved in the regulation of muscle repair via skeletal muscle stem cell activation, connective tissue formation, and regulation of the immune response. TGF-β1 can be produced by multiple lineages of leukocytes and stromal cells, including FAPs and myoblasts. During muscle regeneration after injury, macrophages are the major source of TGF-β1 production [59, 60]. In the inflammatory resolution and regeneration phase, a macrophage subset showed a profibrotic phenotype by increasing its production of TGF-β1 [42, 60, 61]. A recent study also demonstrated that myeloid-specific deletion of *Tgf-b1* abrogated the fibrotic response and reduced FAP proliferation while simultaneously enhancing muscle regeneration in a polytraumatic model of ischemia/reperfusion and CTX that causes extensive fibrosis [60]. FAPs have the potential to differentiate into adipocytes and myofibroblasts, and FAP proliferation and differentiation into myofibroblasts contribute to extracellular matrix (ECM) deposition [62, 63]. Thus, TGF-β1 secreted by myeloid cells is crucial for fibrosis by orchestrating ECM deposition and FAP activation.

## Eosinophils in muscle regeneration

Signals and pathways of the type 2 immune response also appear to be involved in muscle regeneration. For instance, deleting the IL-4 receptor or a combination of the IL-4 and IL-13 receptors resulted in significant delays in regeneration after muscle injury [64]. Additional work has shown that eosinophils rapidly infiltrate into damaged skeletal muscle, activate FAP proliferation by producing IL-4 and, at the same time, inhibit the differentiation of FAPs into adipocytes, thereby promoting muscle regeneration [64].

## Intercellular communication between FAPs, MuSCs, and immune cells

FAPs are a population of muscle-specific mesenchymal stromal cells that exhibit the potential to differentiate into fibroblasts and adipocytes [4, 7]. FAPs express transcription factor 7 like 2 (TCF7L2) and originate from the mesoderm lateral plate in chick and mouse [4, 7, 65]. They also express surface markers, such as platelet-derived growth factor receptor alpha (PDGFRα), stem cell antigen-1 (SCA-1), and cluster of differentiation 34 (CD34), but not PAX7 or any myogenic markers. Previous studies have shown that FAPs are crucial for muscle homeostasis and regeneration. However, they are also the major source of fibroblasts and adipocytes in pathological conditions, such as muscle dystrophy, atrophy and obesity-associated fat infiltration and dysfunction [62].

FAPs are maintained in a quiescent state in uninjured muscle. However, depletion of FAPs by tamoxifen injection into uninjured PDGFRα-creER-DTX mice induces muscle atrophy and decreases the number of MuSCs, indicating that FAPs are required for the maintenance of both skeletal muscle and the MuSC pool under homeostatic conditions [66]. Furthermore, depletion of FAPs resulted in impaired skeletal muscle regeneration accompanied by decreased expansion of MuSCs and CD45^+^ hematopoietic cells after injury [66], suggesting that FAPs closely communicate with MuSCs and leukocytes to respond properly to muscle injury. Activated FAPs also strongly upregulate the expression of WNT1-inducible-signaling pathway protein 1 (WISP1) [67]. WISP1 promotes MuSC expansion and myogenic commitment via asymmetric cell division, which is likely to be required to maintain the local MuSC pool. FAPs also support myoblast differentiation by directly communicating with MuSCs and myoblasts via cytokines, including IL-6 and IGF-1 [4, 64, 68]. In vitro studies have also shown that FAPs promote the proliferation and differentiation of MuSCs [69, 70]. Overall, these findings indicate that FAPs regulate the functions of MuSC through multiple signals.

Additional studies suggest that MuSCs reciprocally regulate FAPs in response to tissue damage. Upon muscle injury, FAPs are activated and start proliferating, which peaks around day 3 post-injury [64, 71]. An in vitro study has indicated that EGF derived from MuSCs activates FAPs and promotes their differentiation to myofibroblasts. Importantly, differentiation of FAPs to adipocytes is inhibited by co-culture with myotubes [7], indicating that interaction with MuSCs is crucial for determining the differentiation fate of FAPs.

Intercellular communication between FAPs and immune cells is also important for muscle regeneration. For instance, the Th2 cytokines IL-4 and IL-13, which are released by eosinophils that are rapidly recruited after muscle injury, are crucial for the activation of FAPs to support myogenesis [64, 71]. Conversely, FAPs can affect immune cells. FAPs are the major source of IL-33, which is a key driver of the accumulation of a subset of Treg cells that express the IL-33 receptor ST2 in injured muscle [72]. Interfering with IL-33/ST2 signaling has been shown to impair regeneration. Moreover, some specific populations of Tregs are involved in regeneration through their interactions with MuSCs. Tregs activated by the IL-33/ST2 axis express amphiregulin (Areg), and Areg directly stimulates EGFR on MuSCs to improve muscle repair [73]. In addition, Tregs are massively recruited to the site of injury and promote the conversion of M1 inflammatory phenotype macrophages to M2 anti-inflammatory phenotype macrophages by releasing IL-4, IL-10, and IL-13 [74]. Interestingly, IL-33-expressing FAPs and Treg cells were reduced in injured muscle in aged mice, suggesting that dysfunctional communication between FAPs and Tregs may cause the regeneration deficiency that occurs with aging.

Muscle regeneration and tissue repair also need to be closely coordinated with remodeling of the ECM [75]. ECM components serve as the physical support for regenerating fibers and influence the retention and activity of secreted mediators in the muscle environment, thereby providing MuSCs with mechanical and biochemical signals [76]. In skeletal muscle, ECM remodeling is essential for maintaining muscle fiber function and skeletal muscle regeneration by regulating MuSC migration and differentiation [77]. It has also been reported that enhancing ECM remodeling via matrix metalloproteinase (MMP)-mediated degradation of ECM components promotes MuSC migration and differentiation [78].

After skeletal muscle injury, FAPs rapidly expand to generate a niche to support myoblast differentiation, and, in parallel, FAP-derived myofibroblasts increase their expression of ECM-related genes that contribute to the creation of an appropriate scaffold for newly generated myofibers. However, prolonged ECM production may cause pathological fibrosis. Lemos et al. reported that pro-inflammatory macrophages induce apoptosis in FAPs by releasing TNF-α, thus promoting the clearance of FAPs that accumulate early after muscle injury [71]. In sharp contrast, TGF-β1 produced by reparative macrophages inhibits TNF-α-induced FAP apoptosis, suggesting that macrophage-derived TGF-β1 may promote fibrosis. Indeed, in *mdx* mice, a model of Duchenne muscular dystrophy, large subpopulations of macrophages express *Tnf* and/or *Tgf-b1*, and blocking TGF-β signaling with nilotinib induced FAP apoptosis and reduced fibrosis in this accelerated fibrosis model [71]. Collectively, these results indicate that the shift in macrophage phenotype from inflammatory to reparative in injured muscle controls ECM remodeling partly by communicating with FAPs [71].

## Vascular endothelial cells promote MuSC proliferation and differentiation

MuSCs are located close to capillaries. Indeed, in cross sections of human deltoid muscle, MuSCs have been shown to have a substantially higher rate of colocalization with capillaries than myonuclei. Furthermore, muscle capillary loss is associated with a decrease in MuSCs in amyopathic dermatomyositis patients [79], suggesting that capillaries are necessary for maintaining a healthy MuSC pool. Based on the close association of MuSCs and capillaries, vascular ECs are another critical cell type in muscle regeneration [2, 79]. In addition to vascularizing regenerating tissues by angiogenesis, vascular ECs provide the MuSC niche itself [79, 80]. Pericytes that envelop capillary ECs may also significantly impact skeletal muscle regeneration, angiogenesis, and fibrosis throughout life [81]. For example, IGF1 and ANGPT1 expressed by pericytes in the myovascular niche induce myogenic cell differentiation and quiescence, respectively [82].

In vitro studies have revealed that ECs promote myogenesis by stimulating the proliferation and differentiation of MuSCs by secreting soluble mediators including IGF-1, HGF, bFGF, PDGF-BB, and VEGF [79]. Conversely, myogenic progenitor cells (MPCs) promote angiogenesis. Latroche et al. found that ECs and MPCs interact to couple myogenesis and angiogenesis. They determined that together with macrophage-derived oncostatin M, EC-derived apelin and FAP-derived periostin control myogenesis/angiogenesis coupling in vitro and are required for myogenesis and vessel formation during muscle regeneration in vivo [6]. They also demonstrated that oncostatin M is induced by IL-4 and IL-10 in macrophages. Collectively, these studies indicate that ECs and MuSCs reciprocally interact to couple angiogenesis and myogenesis in regenerating muscle, with reparative macrophages also contributing closely to this coupling.

## Single-cell RNA sequencing uncovers cellular diversity and intercellular networks during muscle regeneration

During inflammation and tissue repair after injury, the cellular community changes drastically. In addition to the local resident cell population, many cells infiltrate into the wounded area, broadening the cell diversity. Conventional flow cytometry and immunohistochemical analyses have identified the cellular dynamics of multiple cell-types. However, due to the limited availability of known markers and their antibodies, it has been difficult to further scrutinize changes in subpopulations of cells and/or identify unknown cells. Furthermore, bulk RNA-sequencing does not allow for high-resolution analysis of the dynamics of the transcriptome in subpopulations or single cells [3, 83].

Single-cell RNA sequencing (scRNA-seq) analysis, a technology that can obtain gene expression profiles at single-cell resolution in thousands to tens of thousands of cells , has become widely used and has surfaced the diversity and dynamism of cellular populations [84, 85]. It has also enabled us to identify relatively small subpopulations of cells and detect changes in cellular states based on gene expression patterns. Transcriptomic data has also led to the identification of molecular pathways and networks that control cell fate and function. Furthermore, scRNA-seq analyses have provided in vivo evidence supporting models that were established using in vitro and in vivo experiments. For instance, differentiation trajectory analysis of scRNA-seq data was shown to support a model of differentiation of MuSCs to mature myotubes and indicated the sequential expression of transcription factors during this process [86].

Single-cell mass cytometry (cytometry by time of flight [CyTOF]) in combination with scRNA-seq analyses has facilitated the identification of the developmental progression from stem to progenitor cells in skeletal muscle as well as exposed the heterogeneity of MuSCs [87–89]. Two cell surface markers, CD9 and CD104, were identified to distinguish the MuSC population that expresses a high level of *Pax7* from progenitor cell populations expressing *Myf5*, *Myod1*, and *Myog* [87, 88]. MuSCs were shown to express intermediate levels of CD9, but progenitor cells in the early stages of differentiation expressed high levels of CD9 and those in the late stages of differentiation expressed high levels of both CD9 and CD104. Importantly, the combination of these two surface markers were used to identify myogenic progenitors in vivo. This study also revealed a molecular signature (CD44^+^/CD98^+^/MYOD^+^) of the activated stem cell state [87].

Similar cell fate analysis of the MuSC population has also been published in the other reports. Dell’Orso et al. performed scRNA-seq analysis on cells from intact and injured hind limb muscle obtained from 3-month-old C57BL/6 mice and looked at the heterogeneity of the MuSC population. They found that homeostatic MuSCs in uninjured muscle were separated into two clusters: cells in a close-quiescent state and cells in an early-activated state. However, MuSCs obtained from muscle at 60 h post-injury contained 3 populations of cells with distinguished gene expression patterns. One population showed the enrichment of genes involved in ‘muscle contraction’ and ‘myotube differentiation,’ indicating that these cells had started the differentiation process [90]. By looking at the pseudotime single-cell trajectories of homeostatic MuSCs, MuSCs obtained from muscle 60 h post-injury (damaged MuSCs), and primary myotubes, they also showed that dynamic changes in the expression of metabolism genes occurred that were dependent upon differentiation state. Furthermore, mitochondrial gene expression steadily increased during the transitions from homeostatic to damaged MuSCs and then to primary myotubes, which may reflect the increased number of mitochondria in activated and cultured MuSCs [91, 92]. Concordantly, the expressions of genes involved in the glycolysis and TCA cycles as well as the electron transport chain were increased in injured MuSCs and primary myocytes. However, as previously reported, the expression of a subset of genes involved in fatty acid oxidation (such as *Fabp4*, *Pnpla2*, *Acaca*, *Lipa*, *Acsl1*) was high in homeostatic MuSCs but was decreased in activated MuSCs and differentiating myotubes after injury [91]. These results suggest that a shift in cellular metabolism from fatty acid oxidation to glycolysis is observed when MuSCs are activated to proliferate and differentiate.

Evidence of substantial molecular and functional heterogeneity of myogenic stem/progenitor cell populations also emerged in an independent scRNA-seq study [88]. In these experiments, tibialis anterior (TA) muscles of C57BL/6 mice were recovered at 0 (no injury), 2, 5, and 7 days following notexin injection to induce myofiber damage and were analyzed by scRNA-seq. They found that genes expressing syndecans, transmembrane heparan sulfate proteoglycans expressed in muscle precursors during embryonic development and in MuSCs in postnatal skeletal muscle [93], exhibited differentiation stage-specific expression patterns in myogenic stem/progenitor cells and subsequent myogenic populations, indicating the potential of syndecans to be used as markers of differentiation status. The study further classified the MuSC population that uniformly expresses the myogenic surface marker *Itga7* (integrin-a7) into 3 subpopulations: quiescent SCs and cycling and committed myogenic progenitors, which all have heterogeneous expression of syndecan genes. In cycling myogenic progenitors, *Sdc1* (syndecan 1) is highly expressed along with *Ccnb1* (cyclin B1). In contrast, *Pax7* and *Sdc2* are expressed in quiescent SCs and cycling myogenic progenitors but not in committed progenitors.

**Fig. 2:**
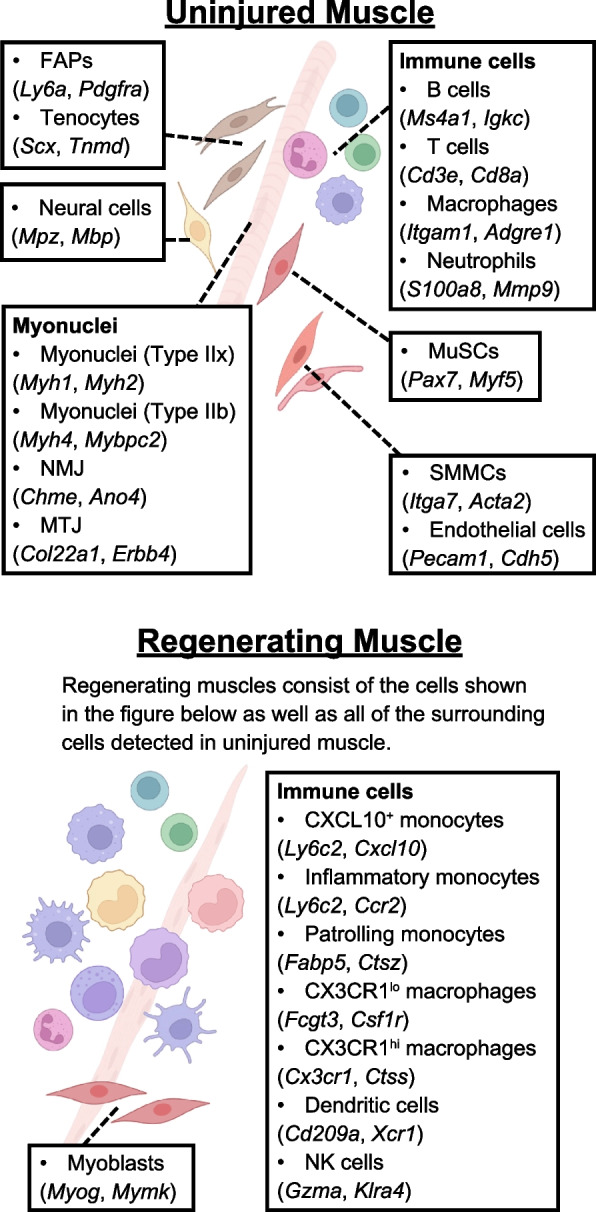
Cellular diversity of skeletal muscle identified by single-cell RNA sequencing. Single-cell RNA sequencing analysis has revealed the detailed cellular composition of adult skeletal muscle and the molecular signature of each mononuclear cell type. This method has not only confirmed the previously established marker-based cell populations (MuSCs, FAPs, immune cells, ECs, pericytes, and neurons) but has also begun identifying new populations of tenocytes and rare cell subpopulations. Further functional analysis will reveal how each of these populations regulates skeletal muscle regeneration, leading to a better understanding of the pathophysiology of sarcopenia and fibrosis caused by regeneration defects. NMJ: neuromuscular junction; MTJ: myotendinous junction; SMMC: Smooth muscle-mesenchymal cell. Created with BioRender.com

Single-cell analysis has also revealed the presence of previously unknown new cell populations in the post-injury muscle stroma. Using a combination of single-cell mass cytometry and single-cell transcriptome analysis revealed the presence of at least 10 different cell populations, including 2 that were previously understudied, in uninjured skeletal muscle in mice [89]. One of newly identified populations is a tenocyte (tendon cell)-like cell population that expresses *Scx* (scleraxis), while the other population consists of smooth muscle-mesenchymal cells (SMMCs) that express *Itga7* (integrin alpha 7) but not *Vcam1* (Fig. 2). These *Scx* expressing cells and SMMCs were suggested to play ancillary roles in skeletal muscle regeneration based on their possible contributions to ECM remodeling and their synergy with MuSCs to form myofibers, respectively.

Additional scRNA-seq analyses have revealed that interstitial cell populations are dynamically altered over the course of injury. After injury, transient increases in the proportions of multiple immune cell types and concomitant declines in the proportions of non-immune cell populations were observed [88]. The immune cell populations that were found to be dramatically increased in the regenerating area after injury include *Cxcl10*^+^ monocytes, inflammatory monocytes, patrolling monocytes, *Cx3cr1*^*lo*^ macrophages, *Cx3cr1*^*hi*^ macrophages, dendritic cells, and NK cells (Fig. 2). These results collectively recapitulated those previously shown in individual FACS analyses. In addition, single-nucleus RNA-sequencing (snRNAseq) has provided detailed gene expression information from myonuclei in myofibers, which are difficult to isolate and analyze by scRNA-seq because of their multi-nucleated structure [94–97]. Using this approach, a previously unrecognized subtype of regenerating myonuclei [97] has been identified (Fig. 2).

Moreover, McKellar et al. curated 23 newly collected scRNA-seq datasets as well as 88 publicly available scRNA-seq and snRNA-seq datasets and compared the results of multiple studies [98]. With their analyses, they successfully annotated the surface markers and transcriptional regulators of muscle stem and progenitor cells to identify those that are specific to the stages of myogenic commitment and myocyte fusion. They also classified novel subpopulations, including three monocyte populations and two macrophage populations that could not be found by FACS analysis, and revealed more complex progenitor cell states and cell-cell interactions in muscle injury [98].

Similar to MuSCs, scRNA-seq studies have also revealed differentiation lineages of FAPs. By integrating long-term, multi-timepoint datasets from day 0.5 to day 21 after skeletal muscle injury, new FAP subpopulations in regenerating skeletal muscle have been discovered [99]. FAPs were identified as cells expressing *Pdgfra*, *Atxn1* (*Sca1*), and *Cd34* as surface markers and were clustered into two populations, *Dpp4*^*+*^ FAPs and *Cxcl14*^*+*^ FAPs, in uninjured muscle. Upon muscle damage, FAP populations with activated transcriptional characteristics and enhanced expressions of *Cxcl5*, *Cxcl3*, *Ccl7*, and *Ccl2* were detected. At 3.5–5 days after injury, these activated FAPs changed into a *Wisp1*^+^ subpopulation that was enriched in ECM-remodeling factors. At 10 days after injury, *Dlk1*^*+*^ FAPs were identified, and at 21 days post-injury, *Osr1*^*+*^ FAPs and a fibroblast population (enriched for genes encoding type I collagen) became dominant.

Human skeletal muscle has also been subjected to single-cell analyses [100–102], and these human studies have also discovered diverse cell subpopulations of MuSCs and FAPs. Although analysis of the regenerative process over time is difficult in human skeletal muscle, a series of scRNA-seq analyses of muscle tissues obtained from different pathological conditions may lead to a better understanding of the molecular mechanisms of human skeletal muscle regeneration.

## Conclusion

This review describes the regulatory mechanisms of regenerating skeletal muscle, focusing on the roles of multiple cells, including macrophages, ECs and FAPs, in the activation, proliferation, and differentiation of MuSCs. While it is now clear that a variety of cells contribute to muscle regeneration, much less is known about the functions of these cells in pathological conditions in which skeletal muscle regeneration is chronically impaired, such as muscular dystrophy and other intractable muscle diseases and age-related muscle weakness (sarcopenia). Future studies utilizing aged mice and muscular dystrophy models will help elucidate the cells and their cell-mediated mechanisms that are critical for muscle atrophy.

Since MuSCs are essential cells for muscle regeneration, therapeutic strategies targeting them have been developed for muscle diseases. However, transplantation of MuSCs in humans has not been as effective as expected, and there is a need to establish new therapeutic strategies. Single-cell technologies are accelerating our understanding of the cellular network that regulates tissue regeneration. Indeed, future studies using these rapidly advancing technologies will lead to the development of therapeutic strategies targeting the cell-cell interactions required for proper skeletal muscle regeneration.

Species differences are important issues when examining pathological conditions [103]. Much of our knowledge is based on studies using mouse models, so it is not immediately clear how many of these findings can be applied to human biology and pathology. For example*,* the inflammatory cytokine IL-1β has a negative effect on MuSC growth in humans but has a positive effect on MuSC growth in mice [104]. Therefore, considering the application of therapeutic methods targeting cells that control muscle regeneration, including macrophages, we need to further elucidate the mechanism of muscle regeneration in humans. Using combinations of pluripotent stem cells, such as human iPS cells and organoids consisting of multiple cell types, will help to establish human model systems that recapitulate human muscle biology and regeneration, which will further our knowledge of cell-cell communication in regenerating skeletal muscle in humans.

## Data Availability

N/A.

## Abbreviations

CCR2: C-C chemokine receptor type 2
CCL2: C-C motif chemokine ligand 2
scRNA-seq: Single-cell RNA sequencing
snRNA-seq: Single-nucleus RNA sequencing
SMMC: Smooth muscle-mesenchymal cell
MuSC: Muscle stem cell
FAP: Fibro-adipogenic progenitor
EC: Endothelial cell
ECM: Extracellular matrix
TA: Tibialis anterior
CyTOF: Cytometry by time of flight
